# Intersecting Pathologies: *COL1A1*-Related Syndrome in the Setting of Childhood-Onset Hypopituitarism: Case Report and Literature Review

**DOI:** 10.3390/diagnostics15192453

**Published:** 2025-09-25

**Authors:** Oriana-Eliana Pelineagră, Ioana Golu, Adela Chiriţă-Emandi, Melania Balaş, Nicoleta Ioana Andreescu, Cătălin Vasile Munteanu, Daniela-Georgiana Amzăr, Iulia Plotuna, Diana Aruncutean, Mihaela Vlad

**Affiliations:** 1 2nd Department of Internal Medicine–Discipline of Endocrinology, “Victor Babes” University of Medicine and Pharmacy, P-Ta Eftimie Murgu 2, 300041 Timisoara, Romania; oriana.pelineagra@umft.ro (O.-E.P.); balas.melania@umft.ro (M.B.); amzar.daniela@umft.ro (D.-G.A.); iulia.plotuna@umft.ro (I.P.); vlad.mihaela@umft.ro (M.V.); 2 Department of Endocrinology, County Emergency Hospital Timisoara, Blvd. Liviu Rebreanu 156, 300723 Timisoara, Romania; diana.aruncutean@student.umft.ro; 3 Center for Molecular Research in Nephrology and Vascular Disease, “Victor Babes” University of Medicine and Pharmacy, P-Ta Eftimie Murgu 2, 300041 Timisoara, Romania; 4 Part of ERN ITHACA, Regional Center of Medical Genetics Timis, Clinical Emergency Hospital for Children “Louis Turcanu”, 300723 Timisoara, Romania; adela.chirita@umft.ro (A.C.-E.); andreescu.nicoleta@umft.ro (N.I.A.); catalin.munteanu@umft.ro (C.V.M.); 5 Department of Microscopic Morphology, Genetics Discipline, Center of Genomic Medicine, “Victor Babes” University of Medicine and Pharmacy Timisoara, 300041 Timisoara, Romania

**Keywords:** COL1A1, connective tissue disorder, Ehlers–Danlos syndrome, C1ROD, hypopituitarism, fractures

## Abstract

**Background**: Type I collagen is the most abundant protein of the extracellular matrix. Pathogenic variants in *COL1A1* or *COL1A2* are classically associated with osteogenesis imperfecta (OI) and Ehlers–Danlos syndrome (EDS). An emerging clinical entity—COL1-related overlap disorder—encompasses individuals exhibiting phenotypic features of both conditions. **Methods**: We report a 55-year-old male presenting with disproportionate short stature, grayish-blue sclerae, multiple fractures, long bone deformities, joint hypermobility, and atrophic surgical scarring. The patient also had long-standing, untreated childhood-onset hypopituitarism. Imaging studies revealed numerous prior fractures, bowing of forearm bones, and multiple Wormian bones. **Results**: Genetic testing confirmed a novel heterozygous *COL1A1* exon 14 variant (c.940G > A, p.Gly314Arg), presenting with a phenotype consistent with a COL1-related overlap syndrome. **Conclusions**: This case expands the phenotypic spectrum of *COL1A1* mutations and supports the concept of COL1-related phenotypic overlap.

## 1. Introduction

Osteogenesis imperfecta (OI) is a genetically inherited connective tissue disorder preponderantly characterized by increased bone fragility, recurrent fractures, short stature, and skeletal deformities. Further and inconstant additional features such as blue sclerae, hearing loss, Wormian bones, and dental abnormalities may also be present, proving valuable clinical diagnostic clues [1,2]. To date, 19 distinct subtypes of OI have been described, with types I through IV being the most prevalent (OI type I OMIM #166200, OI type II OMIM #166210, OI type III OMIM #259420, OI type IV OMIM #166220). The clinical phenotype varies depending both on the subtype itself and on the particular genetic variant. However, genotype–phenotype correlations are not absolute: considerable phenotypic heterogeneity may sometimes be observed among relatives carrying the same molecular defect. About 85 to 90% of reported cases are attributed to a disease-causing variant in the *COL1A1* or *COL1A2* gene, most commonly missense variants occurring after the substitution of glycine with another amino acid within the repetitive amino acid triplet Gly-X-Y found in the collagen triple helix domain. Disease severity spans from mild to lethal, with type I OI patients frequently exhibiting milder forms of disease with fewer bone fractures and less deforming skeletal abnormalities while type II OI patients have rather early lethal outcomes, with fractures showing as early as the 14th week of gestation [1,3,4,5].

Ehlers–Danlos syndrome (EDS) comprises a heterogeneous group of connective tissue disorders characterized by diverse clinical manifestations including joint hypermobility, skin hyperextensibility, spine deformities, and atrophic scarring. A recent classification delineates 13 different EDS subtypes associated with at least 20 genes including *COL1A1*, *COL1A2*, *COL3A1*, and *COL5A1* [6]. Disease-causing variants in type I collagen gene have been identified as a genetic cause for Caffey disease (MONDO:0007244), osteogenesis imperfecta (MONDO:0019019), and *COL1A1*-related Ehlers–Danlos syndrome (MONDO:0100599). *COL1A1* was first associated with autosomal dominant *COL1A1*-related Ehlers–Danlos syndrome in 1968 [7]. This group of connective tissue disorders is characterized by hyperextensible skin, impaired wound healing, and joint hypermobility, which may be accompanied by congenital hip dislocation, pronounced joint laxity, and clinical features of OI [1,6,8].

Individuals exhibiting phenotypes with overlapping features of both diseases have been previously documented and initially labeled as OI/EDS overlap syndrome. More recent publications emphasize the use of “COL1-related syndrome” or “COL1-related overlap disorder” as terms to define these patients, as current diagnostic criteria might lead to misclassification in these individuals [1]. Patients with COL1-related syndrome have been initially reported as having mainly EDS-like manifestations alongside characteristic OI features such as blue sclerae or recurrent fractures, while concurrently harboring a variant in the *COL1A1* or *COL1A2* gene. Initially, most variants (missense and splicing) associated with C1ROD were reported in the *N*-terminal helical domain, leading to helix unfolding and its delayed cleavage [3,9,10,11]. Recent reports including patients with various variants hypothesized the existence of genotype–phenotype correlations, suggesting that *COL1A2* variants are more frequently associated with EDS-like phenotype, whereas *COL1A1* variants tend to present with a clinical picture more consistent with OI [4].

We herein present a 55-year-old male diagnosed with COL1-related overlap disorder, confirmed by a likely pathogenic *COL1A1* variant (c. 940G > A, p. Gly314Arg), in the context of long-standing untreated childhood-onset hypopituitarism and osteoporosis of multifactorial etiology. Furthermore, we portray a review of the literature including other patients with overlapping phenotypes that harbor *COL1A1* variants.

## 2. Case Presentation

### 2.1. Initial Presentation

A 55-year-old male presented with complaints of severe hip pain and inability to bear weight on the left lower limb, reporting symptom onset following a twisting motion, without recent trauma. Radiographic investigations revealed a fracture of the left femoral neck. Following the patient’s admission to the Orthopedic Department, an endocrinologic evaluation was requested due to the suspicion of osteoporosis in a relatively young male.

On clinical examination our patient presented with short stature (155 cm) relative to the calculated mid-parental height (171.5 cm), with disproportionate body segments. Body measurements were within normal limits except for a shorter torso and discrepant upper limb length (Table 1). Distinctive craniofacial features included low-set, prominent ears, grayish-blue sclerae, micrognathia, short neck, and occipital flattening. Additional findings comprised the absence of facial and body hair, slightly pale, dehydrated skin, gynecomastia, pectus carinatum, elbow joints deformities, and multiple atrophic scars on the right upper arm and right tight from previous surgeries.

According to the patient’s history, partial hypopituitarism was diagnosed at the age of 9 years; however, no medical records were available to clarify which hormonal pathways were involved. The patient denied a confirmed diagnosis of growth hormone deficiency but reported abnormal puberty and being diagnosed with central hypothyroidism at the age of 18, when L-Thyroxine replacement was recommended. Secondary adrenal insufficiency was diagnosed at a later stage, and treatment with Prednisone was advised. However, the patient reported only intermittent use of both Prednisone and L-Thyroxine, along with poor adherence to scheduled medical follow-ups. At the time of the admission, he was not following any hormone replacement therapy regimen. Further inquiry uncovered that he was born with normal birth weight but slightly reduced length and presented congenital bilateral radial head and hip dislocation, the latter being corrected during early childhood. Dental eruption was delayed, characterized by dental malpositions and discolored teeth. The patient reported delayed developmental milestones, with assisted walking beginning at the age of two. After the age of 17, he experienced five fractures resulting from relatively minor trauma, located in the right femur, left radius, and left tibia, as well as recurrent fractures of the right humerus. The patient also recalled joint hypermobility during adolescence and early childhood, evidenced by the ability to bend the thumb to the forearm, hyperextension, and increased external rotation of the ankle joint, along with mild skin extensibility on the dorsal hands or scalp. He denied fragile or translucent skin and easy bruising. Family history was unremarkable, with normal stature and absence of recurrent fractures or hypermobility in both parents and his sister.

In the setting of the recent femoral neck fracture, we suspected the presence of osteoporosis, which was confirmed by Dual X-Ray Absorptiometry (DXA) demonstrating a total T-score of −4.4 SD in the lumbar spine and −5.3 SD in the hip. Corresponding Z-scores of −3.9 SD and −4.9 SD, respectively, suggested a secondary etiology.

Hormonal assays performed upon admission were consistent with partial hypopituitarism, demonstrating decreased serum cortisol, follicular stimulating hormone (FSH), luteinizing hormone (LH), insulin-like growth factor-1 (IGF-1), and growth hormone (GH) together with a slightly elevated thyroid-stimulating (TSH) with low free thyroxine (FT4) and normal prolactin levels (Table 2). During admission, total hip arthroplasty was recommended for the diagnosed fracture.

### 2.2. Further Investigations

Later that year, he was admitted to our clinic for comprehensive evaluation. To determine the etiology of hypopituitarism, pituitary imaging using magnetic resonance imaging (MRI) was advised; however, the patient presented a contraindication due to the presence of multiple osteosynthesis implants of various ages and unknown composition. Consequently, enhanced computed tomography (CT) was performed, revealing a normal sellar region, without detectable abnormalities, alongside evidence of old fracture lines, and the presence of 10 to 12 Wormian bones was noted.

**Figure 1:**
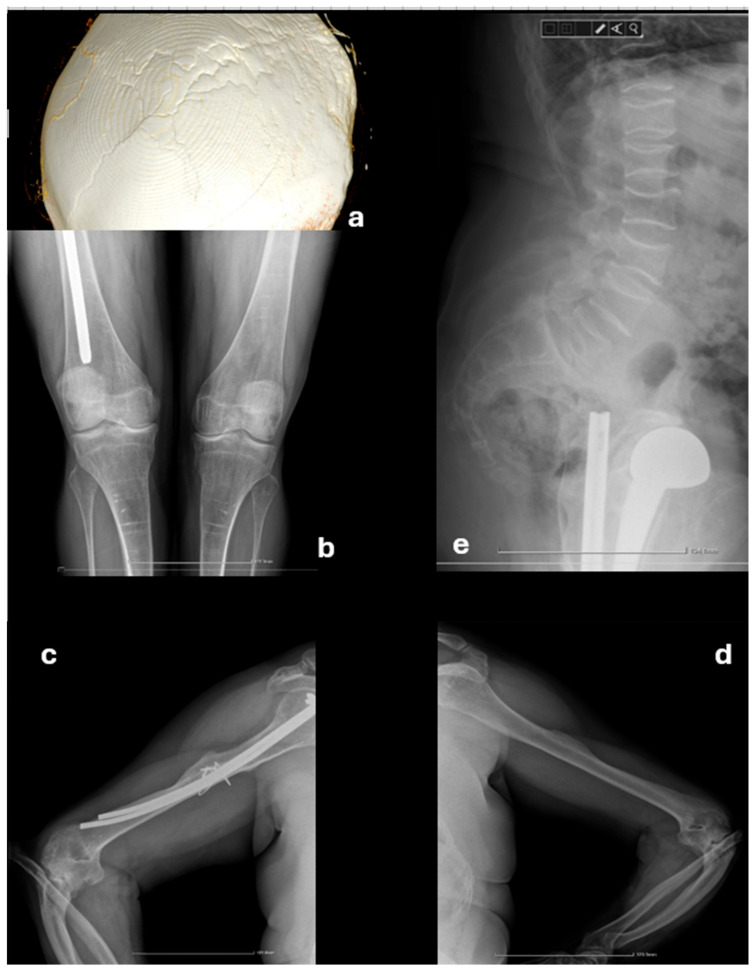
Radiographic investigations. (**a**) Wormian bones. (**b**) Bilateral patella alta and osteosclerotic bands in the femoral and tibial diaphysis. (**c**,**d**) Bilateral radial head dislocation. (**e**) Codfish vertebrae in the thoraco-lumbar spine.

Additional imaging of the elbows, ribs, spine, and long bones was performed to assess previous fracture sites and identify other, previously omitted lesions. The entire spine exhibited a generalized reduction in vertebral height, with a minimum of 6 mm in the cervical and 7 mm in the thoracic spine, accompanied by a codfish vertebrae appearance and dextroscoliosis. Previously unrecognized old fractures were noted on the sixth, seventh, and eighth rib on the left side, as well as the ninth rib on the right side. Persistent radial head dislocation was observed bilaterally, along with bowing deformities of the forearm bones. Horizontal osteosclerotic bands were present in the diaphysis and metaphysis of the distal femur and proximal tibia. Additionally, bilateral patella alta and slight hyperextension of the knee on profile imaging were documented (Figure 1).

Ophthalmic examination was unremarkable except for grayish blue sclerae, but audiological assessment identified bilateral moderate-to-severe neurosensorial hearing loss with a peak threshold of 40 decibels (dB) at a frequency of 250 Hz and a nadir of 85 dB on frequencies above 4000 Hz. Bilateral hearing aids were therefore recommended. Echocardiography demonstrated a normal left ventricular ejection fraction of 55%, an ascending aorta diameter of 37 mm, and mild aortic regurgitation. Cervical ultrasound revealed an enlarged thyroid gland with a volume of 28 milliliters. The hypoechoic, heterogeneous parenchyma combined with elevated anti-thyroperoxidase antibody titers confirmed the diagnosis of Hashimoto’s thyroiditis.

After excluding other causes of low bone mineral density beside hypopituitarism, suspicion of a connective tissue disorder arose due to the constellation of symptoms, including the presence of grayish blue sclerae, distinctive facial features, recurrent fractures, and a history of hypermobility. The Beighton score was calculated to evaluate the severity of hypermobility, scoring 4 out of 9 points. Hyperextension of the fifth finger exceeded 90 degrees, and knee hyperextension reached above 10 degrees. However, our patient could not extend his elbows beyond 10 degrees due to radial head dislocation, nor could he place his palms flat on the ground because of the shorter torso. Multiple atrophic scars from previous surgeries were present, but skin showed no increased extensibility at the time of the examination.

Genetic testing, performed using a commercial NGS gene panel of 4813 genes (TruSight One, Illumina, San Diego, CA, USA) on a MiSeq platform (Illumina, USA) at the Center of Genomic Medicine, “Victor Babeș” University of Medicine and Pharmacy Timișoara, Romania, revealed a likely pathogenic missense variant in exon 14 of *COL1A1* gene (OMIM #120150), NM_000088.4:c.940G > A, NP_000079.2:p.(Gly314Arg), ClinVar Accession: SCV006308727.1 Variation ID:4075334. This variant is located in a genomic region with low tolerance for missense variants and is predicted to produce a glycine-to-arginine substitution within the alpha-1 collagen chain, a known mechanism for *COL1A1*-related disorders. This missense variant is not present in the presumably healthy population (gnomAD v4.1.0), while in silico predictions strongly support its pathogenicity (REVEL score of 0.996). The genomic variant c.940G > A was previously reported in the literature in a patient with a clinical presentation compatible with osteogenesis imperfecta type IV, without echocardiographic or pulmonary function test abnormalities, and a reported age of onset of 14 years [12]. This variant is likely to have occurred de novo in our patient, but genitors were not available for genetic testing.

Considering the patient’s clinical description, personal history, radiological findings, and the *COL1A1* variant, the diagnosis could be best framed as combined osteogenesis imperfecta and Ehlers–Danlos syndrome 1 (OMIM 619115) or Ehlers–Danlos syndrome arthrochalasia type 1 (OMIM 130060). Alternatively, the umbrella MONDO:0100599 code *COL1A1*-related Ehlers–Danlos syndrome could be used in this case.

### 2.3. Management and Follow-Up

**Figure 2:**
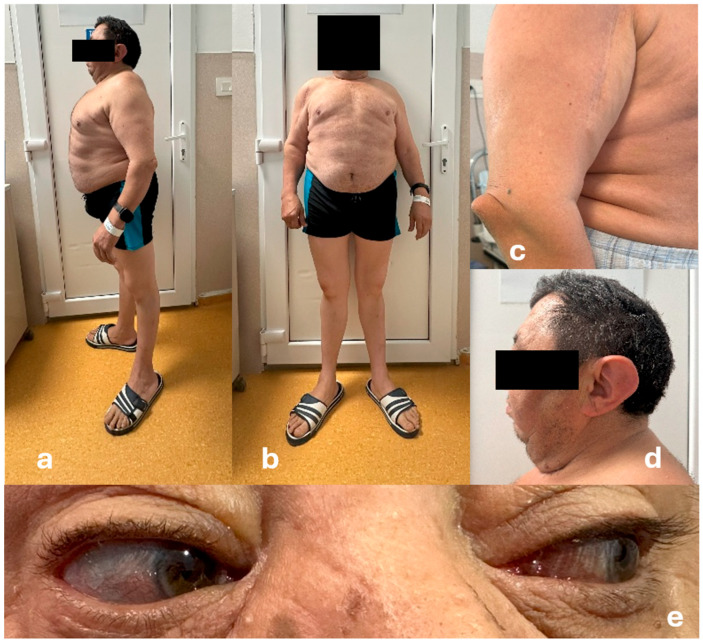
Present clinical aspects after replacement therapy: (**a**,**b**) general appearance, (**c**) atrophic scar upper arm and radial head dislocation, (**d**) prominent low-set ears, (**e**) grayish-blue color of sclerae.

Replacement therapy was started with preoperative, parenteral stress-doses of hydrocortisone, followed by initiation of L-Thyroxine therapy after five days. The patient underwent successful total hip arthroplasty and was discharged with normal blood pressure, as well as normal serum sodium and potassium levels. Maintenance hormone replacement therapy comprising 5 mg of Prednisone was prescribed. The L-Thyroxine dose was gradually increased to 100 mcg daily to maintain serum free T4 within the mid to upper reference range. Testosterone replacement therapy was initiated after admission using Nebido (Testosteronum undecanoate) via intramuscular injection every 3 months. Gynecomastia regressed and male secondary sexual characteristics improved with therapy (Figure 2). This treatment continued until the most recent evaluation in July 2025 when testosterone replacement was discontinued due to erythrocytosis and elevated serum hematocrit levels > 54%. Anti-osteoporotic treatment included calcium and vitamin D supplements in the form of alpha-calcidolum, alongside bisphosphonates therapy since 2022 with zoledronic acid, which led to an improvement in T-scores and bone mineral density (BMD) (Table 3).

## 3. Discussion

Connective tissues disorders are typically diagnosed in the first years of life, guided by characteristic clinical manifestations and the emergence of associated comorbidities such as recurrent fractures or cardiovascular abnormalities [1,13,14]. While early recognition aids disease management and its complications, diagnostic challenges may arise in individuals with subtle phenotypes or overlapping syndromic presentations. Both OI and EDS are heritable connective tissues disorders affecting type I collagen. OI is primarily characterized by bone fragility and progressive skeletal deformities ranging from mild to perinatally lethal forms. In contrast, joint hypermobility and skin hyperextensibility are the hallmark features of most EDS subtypes [3,15]. Intrafamilial phenotypic variance has been previously reported, with individuals of the same family harboring the same pathogenic variant exhibiting different disease severities or incomplete phenotypic expression [2,16]. Notably, some patients may display clinical features for both disorders despite the presence of a variant previously associated with only of the diseases. Individuals with such presentations, involving mixed characteristics of both OI and EDS in the context of a COL1 gene variant, have been recently recognized as constituting a distinct clinical entity referred to as COL1-related overlap disorder (C1ROD) [8].

A comprehensive literature review of similar cases was performed by using the search terms “OI/EDS overlap syndrome”,”COL1-related disorders”, “Overlap syndrome”, and “C1ROD” within PubMed database. Relevant articles were identified and systematically screened. Duplicate entries, as well as reports involving patients without a confirmed *COL1A1* variant, were excluded. The clinical phenotypes, as well as the corresponding genetic variants of included cases, are summarized in Table A1 (Appendix A).

Based on our review of the literature regarding individuals with *COL1A1* variants and confirmed C1ROD, we observed traits such as blue sclerae, joint hypermobility, and fractures as the most frequently encountered clinical manifestations. Unlike classic OI, cases of C1ROD demonstrated a broader spectrum of sclera hue, ranging from grayish to dark blue in reported cases, yet remaining the most common feature in patients with overlap disorder. A history of fractures was documented in approximately 65% of reported cases, although multiple fractures and recurrence appeared to be less frequent events compared to OI patients. Joint hypermobility was present in most patients while other features of EDS were uncommon findings. Notably, pathogenic variants in *COL1A1* have also been recognized as a cause of a rare form of EDS—the arthrochalasia subtype characterized by severe joint hypermobility and bilateral congenital hip dislocation. While hypermobility was frequently reported among C1ROD patients, severity could not be reliably assessed due to inconsistent reporting and lack of standardized scoring in currently available reports. Importantly, similar to our case, bilateral congenital hip dislocation was identified in four of the reported cases while radial head dislocation was present in a single individual. Dental abnormalities or dentinogenesis imperfecta and facial dysmorphism were found only sporadically and appeared to be incidental findings rather than defining features, suggesting the possibility of additional mechanisms leading to their development. Additionally, hearing loss, a characteristic feature of OI, was reported in 11% of included cases. Our current findings are consistent with previously reported phenotypic variability among patients with C1ROD caused by variants in *COL1A1* and *COL1A2*, respectively, with patients harboring *COL1A1* variants exhibiting a spectrum of disease more closely resembling OI rather than EDS, albeit with milder severity [4].

In OI, previous studies have proposed a correlation between clinical severity and the location of the pathogenic variant within the affected gene, particularly in relation to aberrant translation or prolonged processing of the protein [15]. Variants located within the triple helical domain of the type I collagen have been associated with delayed collagen folding due to additional hydroxylation or glycosylation. In addition to positional effects, the specific type of amino acid substituted in the sequence plays a crucial role in determining phenotypic expression, influenced by factors such as its charge or the presence of branched side chain. Glycine, the most frequently substituted amino acid, is essential for triple helix stability due to its small size which permits the tight packing of collagen chains. Previous studies have suggested that variants occurring at the same codon but involving different amino acid substitutions can lead to a spectrum of clinical outcomes, with valine, arginine, and aspartic acid often being associated with more severe phenotypes [8,15]. To further delineate the phenotypic variability observed in C1ROD, it is essential to consider the structural domain affected by the variant. Specifically, pathogenic variants within the *N*-terminal helical domain—referred to as the *N*-anchor and comprising the first 85 residues—have been previously identified as the most frequent variants leading to C1ROD [3,10]. A current review of the literature identified 19 cases out of the total that presented variants in the above-mentioned coding region of *COL1A1*. When compared to the individuals with variants located more distally within the helical domain, patients with N-anchor variants exhibit a higher prevalence of joint hypermobility (94.7% compared to 76.7%), joint dislocations (42.1% compared to 30.2%), and skin hyperextensibility (42.1% compared to 13.9%), resembling a phenotype similar to EDS with sporadic reports of fractures and osteopenia. In contrast, patients harboring variants beyond the first 85 amino acids show a higher frequency of fractures (74.4% compared to 42.1%), dental abnormalities (25.5% compared to 0.5%), and facial dysmorphism (20.9% compared to 1%), features more characteristic of osteogenesis imperfecta. This phenotype stratification supports earlier observations linking *N*-terminal helical domain variants with a milder EDS-like presentation in patients with C1ROD.

In addition to our current case, five other reports have described pathogenic variants located within exon 14 of the *COL1A1* gene, further contributing to the emerging understanding of genotype–phenotype correlation [13,16]. All five individuals were females and exhibited marked joint hypermobility, with Beighton scores ranging from 6 to 9. Except for one, all had a documented history of joint dislocations and fractures. Cutaneous features were frequently observed with skin hyperextensibility, easy bruising, and atrophic scarring. Blue sclerae were noted in three of the five patients, while hearing loss and dental abnormalities were each reported in two cases. Notably, among those with joint dislocation, two individuals presented with bilateral congenital hip dislocation, highlighting a potential recurrent phenotype feature associated with exon 14 variants. In contrast, our patient presents blue sclerae, moderate joint hypermobility with a score of 4 out of 9, congenital dislocations of both the hip and radial head, recurrent fractures, dental abnormalities, facial dysmorphism, and bilateral hearing loss but notably lacked cutaneous features such as skin hyperextensibility or easy bruising. The overlap in clinical manifestation between our case and previously reported cases supports the initial hypothesis of genotype–phenotype correlations related to the location within the affected gene. However, the absence of cutaneous findings may be attributable to the difference in variant type. Specifically, our patient harbors a glycine to arginine substitution, whereas previously reported cases all involve a X substitution by either leucine or cysteine in the sequence Gly-X-Y, potentially leading to a slightly different phenotype. Additionally, it is worth noting that, to date, no male patients with C1ROD harboring exon 14 variants have been documented, precluding sex-based phenotype comparison in this subgroup. The above-mentioned variant has been previously reported in a case series as a single patient with OI type IV; however, no additional clinical data were available for comparison [12].

To our knowledge, there is no reported association between collagen mutations and pituitary-hypothalamic dysfunction except for two prior reports of OI type I associated with panhypopituitarism secondary to pituitary stalk interruption syndrome. In both cases, OI was diagnosed during early childhood, and the presence of additional clinical features led to the subsequent identification of hypopituitarism [17,18]. Unlike typical cases, our patient received a delayed diagnosis following multiple recurrent fractures, with concomitant presence of pituitary dysfunction significantly complicating the diagnostic process. An earlier diagnosis during childhood might have altered the disease trajectory, potentially reducing fracture burden and allowing for timely initiation of bisphosphonate therapy. The patient’s short stature was initially attributed to the combined deficiencies of growth hormone and thyroid hormones. However, further evaluation revealed additional dysmorphic features, with a short neck, short torso, craniofacial abnormalities, and blueish sclerae hue, that could not be fully explained by the deficiency alone. Enhanced CT excluded pituitary adenomas, traumatic lesions, infiltrative processes, and infectious disease as a cause of hypopituitarism, thereby supporting a predominantly idiopathic origin. No abnormalities of the pituitary stalk were identified on imaging. Furthermore, the patient denied a childhood diagnosis of growth hormone deficiency and reported that central hypothyroidism had manifested around the age of 18. Although his height was below the expected mid-parental height, the normally developed body segments, except for a shortened torso, along with mildly increased thyroid volume and normal cognitive development, argued against the presence of pituitary stalk lesions with early-onset growth hormone deficiency and central hypothyroidism in our case. The patient’s history included multiple fractures, which were initially suspected to be the result of secondary osteoporosis due to early-onset of hypogonadism, supported by a decreased Z-score. Nevertheless, given the unusually early onset of fractures, beginning at 17 years of age, and the constellation of phenotypic features, an underlying genetic disorder affecting bone quality was considered. Treatment consisted of hormone replacement therapy with Prednisone, L-Thyroxine, and testosterone, combined with bisphosphonate therapy using zoledronic acid. After three years of antiresorptive treatment, as well as correction of hypogonadism, bone mineral density increased by 26.6% and no additional fractures occurred throughout the course of treatment.

## 4. Conclusions

This case report highlights the diagnostic complexity of COL1-related overlap disorder (C1ROD), particularly when coexisting with endocrine abnormalities such as hypopituitarism that could lead to similar manifestations such as short stature or hypotonia. The presence of both osteogenesis imperfecta- and Ehlers–Danlos syndrome-like features, along with a *COL1A1* exon 14 variant, underscores the importance of considering C1ROD in patients with atypical skeletal and connective tissue manifestations. Our findings reinforce previously described genotype–phenotype correlations, particularly regarding variants in the N-terminal helical domain, and expand the phenotypic spectrum associated with exon 14 variants. Our report also illustrates the necessity for thorough clinical and molecular evaluation, especially in adult patients with complex, overlapping, or contradictory features that may obscure the underlying diagnosis.

## Figures and Tables

**Table 1:** Anthropometric measurements.

Measurement	Value
Height (cm)	155
Weight (kg)	75
Head circumference (cm)	60.5
Armspan (cm)	153
Wingspan-to-height ratio	0.98
Torso length (cm)	50
Left lower limb length (cm)	99
Right lower limb length (cm)	99
Left upper limb length (cm)	69.5
Right upper limb length (cm)	66

**Table 2:** Laboratory measurements.

Investigation	Value	Reference Range
GH (ng/mL)	<0.05	0.07–2.47
IGF-1 (ng/mL)	18.26	53–215
TSH (mUI/L)	7.26	0.55–4.78
Free T4 (pmol/L)	5.03	11.48–22.70
Free T3 (pmol/L)	3.60	3.54–6.47
Cortisol (mcg/dL)	2.94	5.27–22.45
FSH (mIU/mL)	0.06	1.4–18.1
LH (mIU/mL)	0.02	1.5–9.3
Testosterone (ng/dL)	<7.00	187.7–684.1
Prolactin (ng/mL)	15.92	3.7–17.9
Total serum calcium (mg/dL)	10.9	8.4–10.2
Serum phosphate (mg/dL)	5.1	2.5–4.5
Alkaline phosphatase (U/L)	51	38–126
iPTH (pg/mL)	32.8	18.5–88
25OH Vitamin D (ng/mL)	7.10	>30
Anti-TPO Ab (U/L)	493	0–60

GH—growth hormone, IGF-1—insulin-like growth factor-1, TSH—thyroid-stimulating hormone, T4—thyroxine, T3—triiodothyronine, FSH—follicular stimulating hormone, LH—luteinizing hormone, iPTH—intact parathyroid hormone, Anti-TPO Ab—anti-thyroperoxidase antibodies.

**Table 3:** Bone mineral density dynamics after bisphosphonate treatment.

	At Diagnosis		Present	
Parameter	T-Score (SD)	BMD (g/${cm}^{2}$)	T-Score (SD)	BMD (g/${cm}^{2}$)
Total score lumbar spine	−4.4	0.60	−3.0	0.76
Score L1	−4.5	0.51	−2.8	0.70
Score L2	−4.5	0.59	−2.6	0.80
Score L3	−4.4	0.61	−3.0	0.77
Score L4	−4.1	0.69	−3.2	0.79
Total score hip	−5.3	0.23		
Score femoral neck	−4.5	0.31		

SD—standard deviations, BMD—bone mineral density.

## Data Availability

Supporting data presenting findings of this study are available upon reasonable request.

## Abbreviations

The following abbreviations are used in this manuscript:

25OH vitaminD	25-hydroxyvitamin D
a-EDS	Arthrocalasia Ehlers–Danlos syndrome
Anti-TPO Ab	Anti-thyroperioxydase antibodies
BMD	Bone mineral density
C1ROD	COL1-related overlap disorder
c-EDS	Classic Ehlers–Danlos syndrome
CT	Computed tomography
cv-EDS	Cardiac-valvular Ehlers–Danlos syndrome
dB	Decibels
EDS	Ehlers–Danlos syndrome
FSH	Follicular stimulating hormone
FT3	Free triiodothyronine
FT4	Free thyroxine
GH	Growth hormone
IGF-1	Insulin-like growth factor-1
iPTH	Intact parathyroid hormone
LH	Luteinizing hormone
MRI	Magnetic resonance Imaging
OI	Osteogenesis imperfecta
TSH	Thyroid-stimulating hormone
v-EDS	Vascular Ehlers–Danlos syndrome

## Appendix A

**Table A1:** Literature-reported C1ROD cases harboring mutations in COL1A1 gene.

Case No.	Author	Gender	Joint Hypermobility	Joint Dislocation	Skin Hyperextensibility	Easy Bruising	Atrophic Scars	Fractures	Osteopenia	Blue Sclerae	Short Stature	Dental Abnormalities	Facial Abnormalities	Variant	Location
1	Byers [19]	F	+	+	+	-	-	-	-	+	-	-	+		Intron 5
2	Symoens [20]	F	+(8/9)	-	+	-	-	+	+	+	-	+	-	c.3790A > G p.Met1264Val	Exon 49
3	Cabral [10]	M	+	-	-	-	-	-	-	+	+	-	-	c.588 + 4A > T	skip Exon 7
4		F	+(9/9)	-	-	-	-	-	+	+	-	-	-	c.572G > A p.Gly191Asp	Exon 7
5		F	+(7/9)	-	-	-	-	-	-	+	+	-	-	c.609G > A p.Gly203Val	Exon 8
6		F	+(7/9)	-	-	-	-	-	+	+	+	-	-	c.609G > A p.Gly203Val	Exon 8
7		M	-	-	-	-	-	-	-	-	-	-	-	c.634G > C p.Gly212Arg	Exon 8
8		F	+(5/9)	-	-	-	-	-	+	+	+	-	-	c.761G > A p.Gly254Glu	Exon 11
9		F	+(7/9)	-	-	-	-	-	+	+	+	-	-	c.796G > A p.Gly266Glu	Exon 11
10	Cabral [21]	M	+(2/9)	-	-	-	-	+	+	+	-	-	-	c.3196C > T p.Arg1066Cys	Exon 44
11		M	+(4/9)	-	-	-	-	+	-	+	-	-	-	c.3196C > T p.Arg1066Cys	Exon 44
12		M	+(2/9)	-	-	-	-	+	+	+	-	-	-	c.3196C > T p.Arg1066Cys	Exon 44
13		M	-	-	-	-	-	-	-	+	-	-	-	c.3196C > T p.Arg1066Cys	Exon 44
14	Lund [22]	M	+(9/9)	-	-	+	-	+	-	+	-	+	-	c.3106C > T p.Arg1036Cys	Exon 44
15		F	+(7/9)	-	-	+	-	+	-	-	-	-	-	c.3106C > T p.Arg1036Cys	Exon 44
16		F	+(7/9)	-	-	+	+	+	-	+	-	+	-	c.3106C > T p.Arg1036Cys	Exon 44
17	Malfait [3]	M	+(8/9)	+	+	+	+	+	+	+	+	-	-	c.563G > A p.Gly188Asp	Exon 7
18		M	+(9/9)	+	+	+	+	+	+	+	+	-	-	c.607G > T p.Gly203Cys	Exon 8
19	Vandersteen [23]	M	+(2/9)	-	-	-	-	+	-	+	+	+	-	c.3567delT	Exon 49
20		F	+(7/9)	-	-	-	-	-	-	+	-	-	-	c.643G > A p.Gly215Ser	Exon 9
21		F	+	+	-	-	+	-	-	+	-	-	-	c.643G > A p.Gly215Ser	Exon 9
22		M	-	-	-	-	+	+	-	+	-	-	-		Deletion Exon 11-20
23	Shi [9]	M	-	-	-	-	-	-	-	+	-	-	-	c.3521C > T p.Ala1174Val	Exon 48
24		F	+(3/9)	+	-	+	-	+	+	+	+	-	-	c.3521C > T p.Ala1174Val	Exon 48
25		M	+(5/9)	+	-	+	-	+	-	+	-	+	-	c.3521C > T p.Ala1174Val	Exon 48
26		M	+(5/9)	+	-	-	-	+	-	+	+	-	-	c.3521C > T p.Ala1174Val	Exon 48
27		M	+	+	-	-	-	+	-	+	-	-	+	c.3521C > T p.Ala1174Val	Exon 48
28		F	-	-	-	-	-	-	-	-	-	-	-	c.3521C > T p.Ala1174Val	Exon 48
29	Ackermann [24]	M	-	-	-	-	-	-	-	-	-	-	-	c.3196C > T p.Pro814Leufs * 266	Exon 44
30		F	-	-	-	-	-	+	+	+	-	-	-	c.2522delC p.Arg1066Cys	Exon 37
31		F	+	-	-	-	-	+	+	+	-	-	-	c.3196C > T p.Pro814Leufs * 266; c.2522delC p.Arg1066Cys	Exon 37,44
32		M	+	-	-	-	-	+	+	+	-	-	-	c.3196C > T p.Pro814Leufs * 266; c.2522delC p.Arg1066Cys	Exon 37,44
33		M	-	-	-	-	-	+	-	+	-	-	-	c.3196C > T p.Pro814Leufs * 266; c.2522delC p.Arg1066Cys	Exon 37,44
34	Mackenroth [14]	F *	+(5/9)	-	-	-	-	+	+	-	-	-	-	c4009-1G > A	Intron 49
35		M	+(6/9)	-	-	-	-	+	+	-	-	-	-	c.4009-1G-A	Intron 49
36	Symoens [25]	F	+(3/9)	+	-	+	+	+	-	-	-	-	-	c.3150_3158del p.Ala1053_Gly1055del	Exon 44
37	Lu [26]	M	+(6/9)	+	+	+	-	+	-	+	+	+	+	c.671G > A p.Gly224Asp	Exon 9
38	Lin [2]	F **	+	+	-	+	+	+	-	+	-	-	+	c.2010delT p.Gly671Alafs * 95	Exon 30
39		F **	+	-	-	+	+	+	-	+	-	-	+	c.2010delT p.Gly671Alafs * 95	Exon 30
40		M	-	-	-	-	-	+	-	+	-	-	+	c.2010delT p.Gly671Alafs * 95	Exon 30
41	Morlino [11]	M	+(8/9)	+	+	-	+	+	-	+	-	-	-	c.326G > A p.Gly109Asp	Exon 3
42		M	+(5/9)	+	+	-	-	-	+	+	-	-	-	c.581G > C p.Gly194Ala	Exon 7
43		M	+(5/9)	+	-	-	+	+	-	+	-	-	-	c.2073delT p.Gly692Valfs * 74	Exon 31
44		F	+(6/9)	-	-	-	-	-	-	+	-	-	-	c.2073delT p.Gly692Valfs * 74	Exon 31
45		F	+(7/9)	-	-	-	-	+	+	+	-	-	-	c.1243C > T p.Arg415 *	Exon 19
46		M	+ (4/9)	-	+	+	+	+	-	+	-	-	-	c.670G > A p.Gly224Ser	Exon 9
47	Duong [16]	F	+(9/9)	+	+	-	+	-	-	-	-	-	-	c.934C > T p.Arg312Cys	Exon 14
48		F	+	-	+	-	+	+	-	-	-	+	-	c.934C > T p.Arg312Cys	Exon 14
49	Foy [13]	F	+(6/9)	+	+	+	-	+	+	+	-	+	-	c.935G > T p.Arg312Leu	Exon 14
50		F	+ (7/9)	+	-	+	-	+	+	+	-	-	-	c.935G > T p.Arg312Leu	Exon 14
51		F	+(7/9)	+	+	+	-	+	-	+	-	-	-	c.935G > T p.Arg312Leu	Exon 14
52	Morabito [8]	F	+(7/9)	-	-	-	-	-	+	+	+	+	+	c.3235G > A p.Gly1079Ser	Exon 44
53	Takeda [27]	M	+	-	+	-	-	+	-	+	-	-	-	c.658C > T p.Arg220 *	Exon 9
54		F	+	+	-	+	-	+	+	+	+	-	-	c.571G > T p.Gly191Cys	Exon 7
55	Venable [4]	M	+	+	-	-	-	+	-	+	+	-	-	c.423_433delinsT p.Gly142Aspfs * 120	Exon 5
56		M	-	-	-	-	-	+	-	+	-	-	-	c.1128del p.Gly377Alafs * 164	Exon 17
57		F	+(4/9)	-	-	+	-	+	+	+	-	-	-	c.1587del p.Gly530Valfs * 11	Exon 23
58		F	+(7/9)	+	-	+	+	-	-	-	-	-	-	c.2197C > T p.Arg733Cys	Exon32
59		M	-	-	-	+	+	+	+	+	-	-	-	c.3421C > T p.Arg1141 *	Exon 46
60	Hassib [28]	F	+(3/9)	-	-	-	-	-	+	+	+	+	+	c.4340T > G p.Val1447Gly	Exon 51
61		F	+(3/9)	-	-	-	-	-	+	+	+	+	+	c.1678G > A p.Gly560Ser	Exon 25
62	Nunez-Ordonez [29]	F	+	-	+	-	-	-	-	+	-	-	+	c.572G > A p.Gly191Arg	Exon 7
**Total (feature present/absent)**			**51/11**	**21/41**	**14/48**	**19/43**	**15/47**	**40/22**	**26/36**	**52/10**	**17/45**	**12/50**	**11/51**		

* Additional mutation in TNXB gene c.3637G > A. ** Additional mutation in COL5A1 gene c.5334A > G.
